# Association between Benign Paroxysmal Positional Vertigo and Thyroid Diseases: Systematic Review and Meta-Analysis

**DOI:** 10.1055/s-0043-1769496

**Published:** 2023-09-14

**Authors:** Cyntia Machado Lima, Daniel Felipe Fernandes Paiva, Ana Paula Corona, Marcus Miranda Lessa

**Affiliations:** 1 Postgraduate Program in Health Sciences, Faculty of Medicine, Universidade Federal da Bahia, Salvador, BA, Brazil; 2 Postgraduate Program in Odontology, Universidade de Campinas, Campinas, SP, Brazil; 3 Department of Speech-Language Pathology and Audiology, Universidade Federal da Bahia, Salvador, BA, Brazil; 4 Department of Otorhinolaryngology, Universidade Federal da Bahia, Salvador, BA, Brazil

**Keywords:** benign paroxysmal positional vertigo, hypothyroidism, hyperthyroidism, hashimoto thyroiditis

## Abstract

**Introduction** Benign paroxysmal positional vertigo (BPPV) is the peripheral vestibular dysfunction that most affects people worldwide, but its etiopathogenesis is still not fully understood. Considering the etiological diversity, some studies highlight the association between BPPV and thyroid diseases.

**Objective** To investigate the association between thyroid diseases and BPPV.

**Data Synthesis** Systematic review and meta-analysis of epidemiological studies searched in the PubMed, Web of Science, Embase, Cochrane Library, and Scopus databases. Studies that were fully available and investigated the association between BPPV and thyroid diseases were selected. The articles that composed the meta-analysis were analyzed using the dichotomous model, the Mantel-Haenszel statistical test, odds ratio (OR), and a 95% confidence interval (CI). Of the 67 articles retrieved from the databases, 7 met the eligibility criteria of the systematic review, and 4 had data necessary to perform the meta-analysis. Qualitative analysis revealed that the studies were conducted in the European and Asian continents. The predominant methodological design was the case-control type, and thyroid dysfunctions, hypothyroidism, and Hashimoto thyroiditis occurred more frequently. The meta-analysis showed no association between hypothyroidism and BPPV; however, there was a statistically significant relationship between Hashimoto thyroiditis and BPPV.

**Conclusion** The meta-analysis results suggest a possible association between BPPV and Hashimoto thyroiditis. Nevertheless, we emphasize the need for further studies to elucidate the evidence obtained.

## Introduction

Benign paroxysmal positional vertigo (BPPV) is the peripheral vestibular dysfunction that most affects people in the world, affecting ∼ 2.4% of the population throughout life, with women being more likely to develop the condition, a proportion of 1.68 women for every man, irrespective of the age of the patients. 1 2 3 This dysfunction is characterized by short-term and intense vertigo attacks triggered by a change in head position. 2 3 4 5 In general, the first seizures occur after the age of 49, despite these episodes of vertigo tending to be more frequent in the elderly population. 2 5

Vertigo generates significant damage in various aspects of an individual's life, the most frequent being the decrease in quality of life, reduced autonomy, impact on work function, increase in morbidities, and, especially in the elderly population, the risk of falling from standing height in addition to expenses with diagnostic tests and intervention procedures. 6 7

The etiopathogenesis of BPPV is still not fully understood. Some cases are attributed to head trauma, vestibular neuritis, osteoporosis, hypovitaminosis D, diabetes mellitus, hyperlipidemia, stroke, and thyroid diseases, among others. 4 8 9 Given this etiological diversity, some studies highlight the association between BPPV and thyroid diseases. 10 11 12

According to Wajchenberg et al., 13 thyroid diseases are divided into those that affect the function of the thyroid gland, in which there is an increase or decrease in serum concentrations of thyroid hormones, and those that promote trophic changes in the organ, characterized by a diffuse increase in the gland, development of a nodule or multiple nodules.

Hypothyroidism results from a decrease in the concentration or action of thyroid hormones in the body, causing a decrease in metabolism and bodily functions that depend on thyroid hormones. Its pathological opposite is hyperthyroidism, characterized by increased hormonal components in focus. The leading cause of primary hypothyroidism is Hashimoto thyroiditis (HT), an autoimmune disease associated with thyroid gland inflammation. 13 14 15

Thus, the present study aims to investigate the association between thyroid diseases and BPPV since, due to complications and the high prevalence in the general population, it is necessary to know the predictors of positional vertigo in order to guide conducts that provide preventive actions, as well as a better prognosis of this dysfunction.

## Review of Literature

The present systematic review and meta-Analysis were prepared following the considerations proposed by Honório et al. 16 and filed with PROSPERO under registration number CRD42021267146.

The electronic search engines PubMed, Web of Science (all databases), Embase, Cochrane Library, and Scopus were used from June 25 to August 4, 2021, to obtain the articles on the topic. The descriptors were selected through the Medical Subject Headings (MeSH) *Benign Paroxysmal Positional Vertigo,* *Hypothyroidism* , *Hyperthyroidism* , *Hashimoto* *Disease* , and their corresponding input terms suggested by the MeSH database itself, in order to cover the most significant number of publications. Therefore, the following base strategy was established: ((“ *Benign Paroxysmal Positional Vertigo* ”) AND ( *Hypothyroidism* OR *Hyperthyroidism* OR *Hashimoto* *Disease* )),” being replicated in all search engines, except for EMBASE, in which the MeSH descriptors were replaced by their Emtree descriptors, as suggested by the platform.

The articles included met the following criteria: (1) case-control studies, cohort studies, or clinical trials that investigated the association between BPPV and thyroid diseases, published without restriction as to the year, in English, Portuguese and Spanish; (2) sample composed of individuals with thyroid diseases (hypothyroidism, hyperthyroidism or HT); (3) description of the BPPV diagnosis based on the clinical history of positional vertigo and/or the presence of typical nystagmus in the Dix-Hallpike or Supine Roll test maneuvers; (4) works available in full in the scientific databases researched. Studies without a comparison group and those whose sample consisted of individuals with visual, motor, and/or cognitive impairment were excluded.

In a virtual meeting, two evaluators discussed the inclusion and exclusion criteria to align the selection of articles. A Kappa index of 0.91 of agreement between the examiners was obtained in analyzing titles and abstracts. After a complete reading of the articles in the selection stage, this index was 0.93, demonstrating high reliability in both stages. Differences between them were resolved by consulting a third researcher. Studies that met the selection criteria were fully evaluated. The bibliographic references of the selected articles were also analyzed to identify potentially relevant publications. Those that met the previously established criteria were integrated into the review.

The following information was extracted from the studies: authors, title, year of publication, country, type of study, age group of the groups, number of individuals in each group, gender, and incidence of BPPV in individuals with thyroid diseases, in addition to the type of thyroid pathology reported.

Two tools for analyzing the risk of bias were used, the Cochrane Risk of Bias, using RevMan 5.4, and the Newcastle-Ottawa scale, to obtain greater precision in the analysis due to the methodological design of the selected articles.

The Cochrane Risk of Bias tool is considered the gold standard for evaluating randomized clinical trials, sometimes used to analyze other types of methodological design, but with some imprecision. The analyzed domains are the generation of a random sequence of participants, concealment of allocation of sampling units, blinding of participants and professionals, blinding of outcome evaluators, incomplete outcomes, selective reports, and other possible sources of bias previously established by the reviewers. Each item analyzed is classified according to the information in the article as low risk, uncertain risk, and high risk of bias. The risk of bias analysis for an outcome is obtained from the score recorded by most articles. 17

Regarding the item “other sources of bias,” the following criteria were established for the present study: for the diagnosis of BPPV, the presence of both maneuvers (Dix-Hallpike and Supine Roll test) was classified as low risk of bias. A high risk of bias was considered if only one or none of the maneuvers was reported; for the diagnosis of thyroid diseases, a low risk of bias was adopted when performed from a blood test.

The Newcastle-Ottawa scale analyzes the risk of bias in case-control and cohort observational studies. It comprises three domains of risk assessment bias: selection of groups, consisting of four items; comparability of groups, consisting of two items; and exposure/result, consisting of three items. Each analyzed topic receives the value one (1) when it meets the criterion, or zero (0) in the absence of the requirement, being classified according to the scores obtained from 0 to 4 low methodological quality, from 5 to 6 moderate quality, and ≥ 7 high quality. 18 19

In the systematic review, the data were evaluated based on the critical analysis of the findings in each study included in the present review. All outcomes were taken as a basis for the discussion of effects, the main characteristic being the association or not between BPPV and thyroid diseases (hypothyroidism, hyperthyroidism, and Hashimoto thyroiditis).

A meta-analysis was conducted using RevMan 5.4 software. The dichotomous model was used to configure the variables, using the Mantel-Haenszel statistical test and random effect model analysis. Data were analyzed using odds ratio (OR) and their respective 95% confidence interval (CI) values for the included studies and the total of the presented meta-analysis. The OR was chosen because it is a dichotomous study aiming to identify whether thyroid disease is a risk factor for BPPV. Additionally, the included studies had a case-control methodological model, with OR being the measure of deprecated effect.

**Fig. 1:**
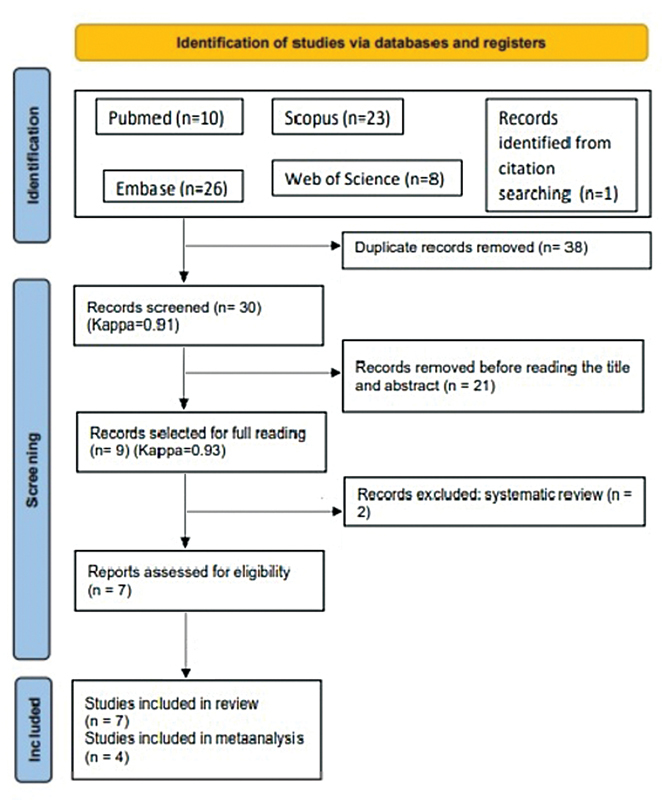
PRISMA flowchart from the identification stage to selecting articles for meta-analysis.

Of the 67 articles retrieved from the databases, 7 met the eligibility criteria of the present systematic review, and 4 had data necessary to carry out the meta-analysis (dichotomous data for thyroid diseases and BPPV), according to the model flow diagram of the PRISMA 20 21 association illustrated in Figure 1 .

The seven articles selected for the qualitative analysis were developed in the European and Asian continents, with three were carried out in Italy ( Table 1 ). The predominant methodological design was the observational case-control type. The participants ranged from 70 to 51,485; the most frequent thyroid disorders were hypothyroidism and Hashimoto thyroiditis. Concerning the bias analysis by the Newcastle-Ottawa scale, the scores ranged between 5 and 6, except for one article with a 4. Table 2 shows the risk of bias obtained through the Cochrane tool, where there is an imprecision in the analysis of the articles.

**Table 1:** Characteristics of the analyzed studies and risk of bias according to the Newcastle-Ottawa scale

Author, year	Country	Type of study	Population	Thyroid dysfunction	BPPV incidence	*p- value*	NOS	Meta-Analysis
HSU, Chiao-Lin et al, 2019 10	Taiwan	P	Total n of the study = 51485	Hypo	RR = 1.26 (0.18–9.02)	0.817	5	NO
				Hyper	RR = 2.46 (1.24–4.87)	0.01		
PAPI, G et al, 2009 10	Italy	CC	TG 132	Thyroiditis	OR = 25.6 (6.0–108.6)	< 0.001	5	YES
			CG = 100	Hypo	OR = 12.9 (3.0–55.8)	< 0.001		
MODUGNO, G C et al, 2000 11	Italy	CS	70	Thyroiditis	27.10%	< 0.01	4	NO
PAPI, G et al, 2010 12	Italy	CC	TG = 200	Thyroiditis	OR = 21.73 (5.1547–91.6190)	< 0.000	6	YES
			CG = 200					
CHOI, H G et al, 2021 12	South Korea	CC		Thyroiditis	OR 1.16 (1.02–1.32)	0.025	5	YES
			TG = 19071	Hypo	OR = 1.26 (1.10–1.37)	<0.001		
			CG = 76284	Hyper	OR = 1.13 (1.02–1.26)	0.023		
YILDIR, O T; KAYA, S; KAYA, F B, 2020 24	Turkey	CC	TG = 84	Hypo	OR = 0.5093(0.1097-2.3652)	0.389	5	YES
			CG = 59					
SARI, K et al, 2015 25	Turkey	C	WITH BPPV = 50	TSH	___	–	5	NO
			WITHOUT BPPV = 52	TPO-Ab		0.729		
			CG = 60	TG-Ab		0.812		

Abbreviations: C, cross-sectional; CC, case-control; CG, control group; CS, case series; Hyper, hyperthyroidism; Hypo, hypothyroidism; NOS, Newcasttle-Ottawa scale; P, prospective; TG, test group; TG-AB, thyroglobulin antibodies; TPO-Ab, thyroid peroxidase antibody; TSH, thyroid stimulating hormone.

**Table 2:** Analysis of the risk of bias in the Cochrane Collaboration

**Fig. 2:**
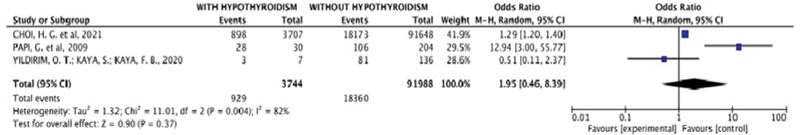
Forest graph of the analysis of the association between hypothyroidism and BPPV.

**Fig. 3:**
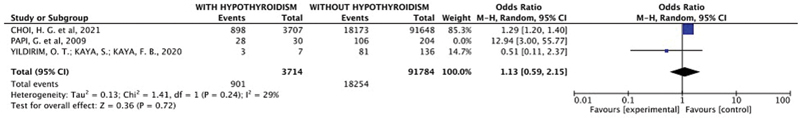
Forest graph for subgroup analysis of the association between hypothyroidism and BPPV, excluding the study by Papi et al. (2009)

Two meta-analyses were performed to carry out the quantitative analysis of the findings. First, we sought to obtain the association between hypothyroidism and BPPV ( Figure 2 ). Based on the data presented, it is observed that hypothyroidism does not present statistical significance (OR = 1.95 [0.11–2.37]), despite being a frequently reported clinical factor. Nevertheless, the first analysis shows high inter-study heterogeneity (I ^2^ = 82%), and a subgroup analysis is recommended for a more accurate assessment. As a result, it was decided to suppress the study by Papi et al. 10 and perform a subgroup analysis between studies with similar methods (I ^2^ = 29%). Still, the result obtained remained without statistical significance (OR = 1.13 [0 .59–2.15]), thus refuting the association between hypothyroidism and BPPV ( Figure 3 ).

**Fig. 4:**
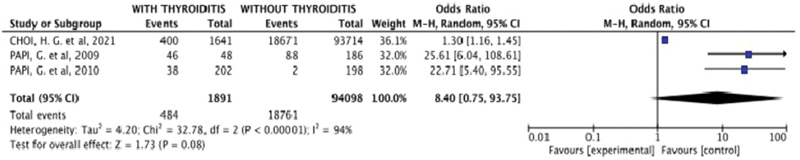
Forest graph of the association analysis between thyroiditis and BPPV.

**Fig. 5:**
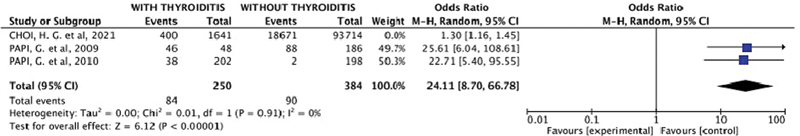
Forest graph of the subgroup analysis of the association between thyroiditis and BPPV, excluding the study by Choi et al. (2021).

The second quantitative analysis investigated the association between Hashimoto thyroiditis and BPPV ( Figure 4 ). According to the data presented, we can infer that there was no statistical significance (OR = 8.40 [0.75–93.75]). However, a high heterogeneity (I ^2^ = 94%) was observed, considering the methodological differences. This time, it was between the study by Choi et al. and other studies that associated thyroiditis with BPPV. So, a subgroup analysis was performed, omitting the divergent article. From the new analysis, the result obtained favored the control group (OR = 24.11 [8.70–66.78]), and the heterogeneity proved to be adequate (I ^2^ = 0.0%), evidencing Hashimoto thyroiditis as a risk factor for BPPV ( Figure 5 ).

## Discussion

Our results indicate a possible association between Hashimoto thyroiditis and BPPV. This relationship was demonstrated through the significance of the OR obtained in the meta-analysis, which, in turn, is in line with the conclusions of Modugno et al., 12 Papi et al., 10 Papi et al 11 ., and Choi et al. 22 According to these authors, a high serum level of antithyroid antibodies, TPOAb and TGAb, increases the risk of developing BPPV, thus supporting the theory that thyroid autoimmunity may favor the development of vestibular diseases, regardless of thyroid function. Chiarella et al. 23 emphasize that changes in TPOAb levels represent a greater risk of developing vestibular dysfunction than in TGAb, besides confirming the importance of antithyroid antibodies. Therefore, the dosage of TPOAb in the diagnostic investigation of patients with BPPV should be prioritized because it presents a higher risk ratio for vestibular diseases.

However, the study conducted by Sari et al. to investigate the association between BPPV and thyroid autoimmunity did not demonstrate a statistically significant difference between the test and control groups in the dosage of antithyroid antibodies (TGAb and TPOAb). It was the only study in the present systematic review with conflicting data with the others. 10 11 12 22

Four studies analyzed the association between hypothyroidism and BPPV. Two of them, Turgay Yildirim et al. 24 and Hsu et al., 25 found no causal relationship. Nonetheless, the results obtained by Papi et al. 10 and Choi et al. 22 suggested a positive relationship between hypothyroidism and BPPV. One of the strands that explain this relationship stems from studies that suggest an association between low levels of thyroid hormones and changes in the cardiovascular system, sufficient to cause a decrease in body blood flow and, consequently, promote a decrease in microcirculation in the inner ear, thus explaining a higher rate of BPPV in people with thyroid disease. 26 27

Meta-analysis data did not demonstrate statistical significance between hypothyroidism and BPPV, in accordance with Hsu et al., 25 Modugno et al., 12 and Papi et al. 10 The findings recorded in Figure 2 demonstrate that the studies by Choi et al. 22 and Papi et al. 10 agree about a positive relationship between hypothyroidism and BPPV. However, the assessment and analysis measures and criteria used by Turgay Yildirim et al. 24 may have influenced the result of the meta-analysis, causing it to focus after the significance line, making such a relationship irrelevant. As a result, we can infer that the results regarding hypothyroidism are still controversial, and more studies are needed to clarify this link.

A meta-analysis of the association between hyperthyroidism and BPPV was not performed due to the small number of studies and the measures and analysis criteria used. Still, two studies 2 4 that made up the systematic review analyzed this relationship and found a risk relationship for developing BPPV in people with hyperthyroidism. This causal relationship probably stems from the fact that people with hyperthyroidism usually develop mild hypercalcemia due to thyroid hormone-mediated bone resorption. As otoconia are basically made up of calcium carbonates, in situations where the concentration of calcium is increased, there will also be a greater amount of this free element in the endolymph, reducing the body's ability to dissolve the displaced otoconia of the otolithic membrane. 23 27

The quality of the studies included in the present systematic review presented a moderate score, ranging from five to six points, according to the NOS scale. The study by Modugno et al. 12 was the only one that presented low methodological quality due to the lack of clarity of the inclusion and exclusion criteria of the samples, lack of details regarding the battery of exams and/or tests included in the evaluation, as well as the maneuvers used for the diagnosis of BPPV. The aforementioned methodological limitations justify the score obtained on the NOS scale and, consequently, the absence of this study in the meta-analysis.

Three studies were excluded from the meta-analysis due to methodological differences regarding the experimental model or the representations of their results that made the analysis through the OR impossible. Hsu et al. 25 performed the analysis through the hazard ratio; Modugno et al. 12 showed only an increase in prevalence in the population studied, but they did not show further specifications about the findings; Sari et al., 8 on the other hand, reported only the standard deviation and mean of BPPV and thyroid diseases in the population studied.

In the articles with the largest number of participants, information was collected from the national health system database from the ICD-10. Several health professionals fed the database, and there were no reports of information recording protocols. 5 7 It is important to mention that the conclusions of these studies considered the influence of thyroid diseases on BPPV, agreeing with much of the researched literature, even with these variables.

The present systematic review and meta-analysis have some limitations. Among them, we can highlight the reduced number of publications that address the relationship between thyroid diseases and BPPV, the lack of standardization for the diagnosis of BPPV, especially considering the blinding of the outcome assessors, as well as the accurate recording of the findings obtained in the Dix-Hallpike and Roll-test maneuvers. Thus, it becomes noticeable that the level of evidence allowed from the current literature, despite indicating a strong trend, is still not sufficient to establish a precise chance relationship.

## Final Comments

The results of the present meta-analysis do not allow establishing an association between thyroid diseases and BPPV, but they reveal evidence of a possible relationship between Hashimoto thyroiditis and BPPV. Therefore, additional studies are needed, using adequate methodological designs, preferably randomized clinical trials, to know and understand the role of thyroid diseases in vestibular dysfunctions.
